# Excision of the Upper Maxillary Bone

**Published:** 1842-12

**Authors:** R. D. Mussey


### ARTICLE V.

*Excision of the Upper Maxillary Bone.* By R. D. Mussey, M. D. &c.

Thomas McGillighan, a locksmith, æt. 22, consulted me, in July, 1839, for a painful affection of the left side of the face, which had existed about eight months. The left nostril was entirely blocked up by an adventitious growth of considerable firmness, which extended anteriorly within half an inch of the margin of the ala and septum, and posteriorly so far as to be felt by the finger above the floating edge of the soft palate. The ceiling of the mouth, on the left side, was pushed downwards, so as to present a slight convexity, and the cheek was more prominent than the other. For the pain which extended along the alveolar arch, he had several teeth extracted, but without any important relief. The general health was not materially affected. As there could be no doubt that the tumour sprung from the antrum, and as its progress had been somewhat rapid, I recommended the excision of the jaw-bone, as soon as the hot weather should subside, and a strict adherence to farinaceous diet, with water and a small quantity of milk for drink, which course was faithfully pursued.

On the 28th of September, 1839, I performed the operation in the following manner.—An incision through the integuments, commencing a quarter of an inch below the tendon of the orbicularis palpebarum, was carried down by the side of the nose, and close to the convex border of the ala, thence horizontally to the median line, from which point the upper lip was cut through vertically. Another curvilinear incision extended from the angle of the mouth to the outer margin of the bony orbit as high as the external canthus. The flap included between these incisions was dissected up and thrown upon the forehead, and the malar bone was exposed by a horizontal incision of an inch backward along the zygoma from the margin of the second incision. An incision on the median line from the incisors to the posterior edge of the hard palate, through the lining of the arch of the mouth, and another through that of the palate, separating it from the palate plate of the palate bone, completed the section of the soft parts. By the aid of a saw and bone nippers the bony connections were divided, and the whole of the upper maxillary bone, except the point of its nasal process—which was left on account of the lachrymal sac—was removed, together with a part of the malar, and the whole of the palate plate of the palate bone. The tumour occupied the cavity of the antrum, had pushed through its anterior wall, and attenuated its flooring, filled up the whole nasal avenue, pressed the septum some way into the right nostril, and crowded itself into the cells of the sphenoid bone, and, if I judge correctly, filled up the whole cavity of the body of that bone. From this situation I dug it out with the point of my finger.

There was not much hemorrhage. Three or four vessels only required the ligature. The flaps were preserved in situ by stitches, and a great part of the wound united by adhesion. No severe pain nor considerable constitutional irritation followed the operation, and on the *tenth day* the patient took a walk in the street. The tumour was firm and somewhat fibrous in some parts, and decidedly encephaloid in others. From its soft and homogeneous texture, I entertained fears that it might return, and enjoined it upon the patient to live without flesh, fish or greasy food, with no condiment except salt, and to drink nothing but water—a course which he has rigidly followed to the present time. He has enjoyed fine health, without a trace of the disease, since the operation—a period of two years and nine months. The winter after the operation, Dr. Cook, an ingenious dentist of our city, inserted a gold palate, with an arch of teeth, which restored a natural appearance to the mouth, and a perfect articulation. This is still worn, and so slight a deviation from symmetry between the two sides of the face exists, that very few would suspect any operation to have been performed upon it.

Mr. McGillighan is now a thriving mechanic, and a worthy citizen.—*Western Lancet.*

---

Science without experience does not get the physician great credit with the patient.—*Ambrose Paré.*

He that would perform any great and noble work, must apply himself diligently to the knowledge of the subject.—*Ambrose Paré.*

It is better to try a doubtful remedy than none at all.—*Celsus.*

